# Broad Ecological Niche in Seashore Lichens Emerges From a Stable, Selective Association With Generalist Algal Symbionts

**DOI:** 10.1002/ece3.72639

**Published:** 2025-12-16

**Authors:** Ivana Černajová, Jana Schmidtová, Ulf Schiefelbein, Francesco Dal Grande, Pavel Škaloud

**Affiliations:** ^1^ Faculty of Science, Department of Botany Charles University Praha Czechia; ^2^ Botanical Garden University of Rostock Rostock Germany; ^3^ Department of Biology University of Padova Padua Italy; ^4^ Centro di Ateneo ‘Orto Botanico’ Padua Italy

**Keywords:** association patterns, ecological amplitude, free‐living algae, metabarcoding, mutualism, salinity gradient, Ulvophyceae, Verrucariaceae

## Abstract

In mutualistic systems, the ability to associate with diverse symbionts of distinct physiological traits often facilitates broadening of the niche. In lichen symbiosis, this process remains understudied. While such flexibility has been demonstrated for some species, others associate only with symbionts of comparable characteristics. We investigated whether the broad niche of *Hydropunctaria*—a genus of seashore lichens inhabiting the supralittoral zone across a salinity gradient—is linked to algal symbiont turnover in response to the changing salinity. Using Sanger sequencing of lichen symbionts and Illumina metabarcoding of free‐living algae, we assessed symbiont diversity, selectivity, and the presence of algal symbionts in the environment. Despite the presence of multiple potential algal partners in the surrounding environment, *Hydropunctaria* exhibited a highly specific and stable association with a single algal symbiont across a wide salinity range. This suggests that the ecological niche breadth in this symbiotic system is driven not by the ability to change partners in response to changing salinity, but rather by the association between generalist, euryhaline symbionts. Our findings also point to selective mechanisms beyond partner availability alone.

## Graphical abstract

In mutualistic symbioses, partner flexibility often broadens the host ecological niche. We found a stable association between littoral lichens and their algal symbionts along a natural salinity gradient, suggesting that the broad ecological niche can be facilitated by association with a single generalist symbiont.

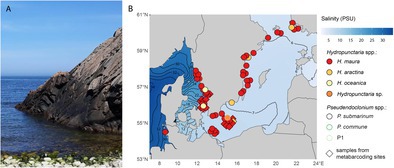

## 1 Introduction

Niche breadth is one of the central concepts in ecology, with implications for species distribution, coexistence, and evolutionary histories, among others. Mutualism has contrasting effects on a host's niche breadth; it may be either reduced due to narrow ecological requirements and/or distribution range of its symbiont (Duffy and Johnson 2017), or it may be extended thanks to symbiont‐mediated tolerance to certain abiotic factors (Peay 2016). Lichens exemplify a mutualistic symbiosis between a lichenized fungus, termed the mycobiont, and an alga or cyanobacterium, termed the photobiont. Lichenized fungi are obligately symbiotic (with exceptions in Ostropales (Wedin et al. 2004)), whereas photobionts occur free‐living as well (Veselá et al. 2024). Therefore, the question shifts to whether or not the partner breadth of the mycobiont (the capacity to associate with multiple symbionts that perform the same role) influences the niche breadth of the holobiont.

Mutualists typically associate with multiple partners—for example, in plant‐pollinator interactions, mycorrhizae, or the human gut microbiome (Schluter and Foster 2012; Voller et al. 2024; Waser et al. 1996)—while coevolution leading to specialization on a single partner, as in yuccas and their monophagous yucca moth pollinators (Pellmyr 2003), is generally rare (Hembry and Althoff 2016). Association with multiple partners might be a mechanism for the expansion of distribution range (Trøjelsgaard et al. 2015) or ecological niche—for example, reef‐building coral species that associate with diverse *Symbiodinium* lineages inhabit a broader range of depths (Baker 2003), while native grasses can colonize coastal or geothermal habitats when associated with specific endophytic fungi (Rodriguez et al. 2008). Similarly, most lichenized fungi associate with multiple photobiont lineages, yet these photobionts typically share similar environmental requirements (Kaasalainen et al. 2021; Peksa et al. 2022; Rikkinen et al. 2002; Škvorová et al. 2022). The ability to associate with photobionts of distinct ecophysiological preferences may have a substantial impact on the lichen's niche breadth, expanding its overall climatic niche and distribution range (Romeike et al. 2002; Blaha et al. 2006; Leavitt et al. 2013; Engelen et al. 2016; Magain et al. 2017; Rolshausen et al. 2018, 2020; Vančurová et al. 2018) or enabling colonization of diverse substrates (Juriado et al. 2019) and heavy‐metal‐polluted soils (Osyczka et al. 2021; Vančurová et al. 2018).

**FIGURE 1:**
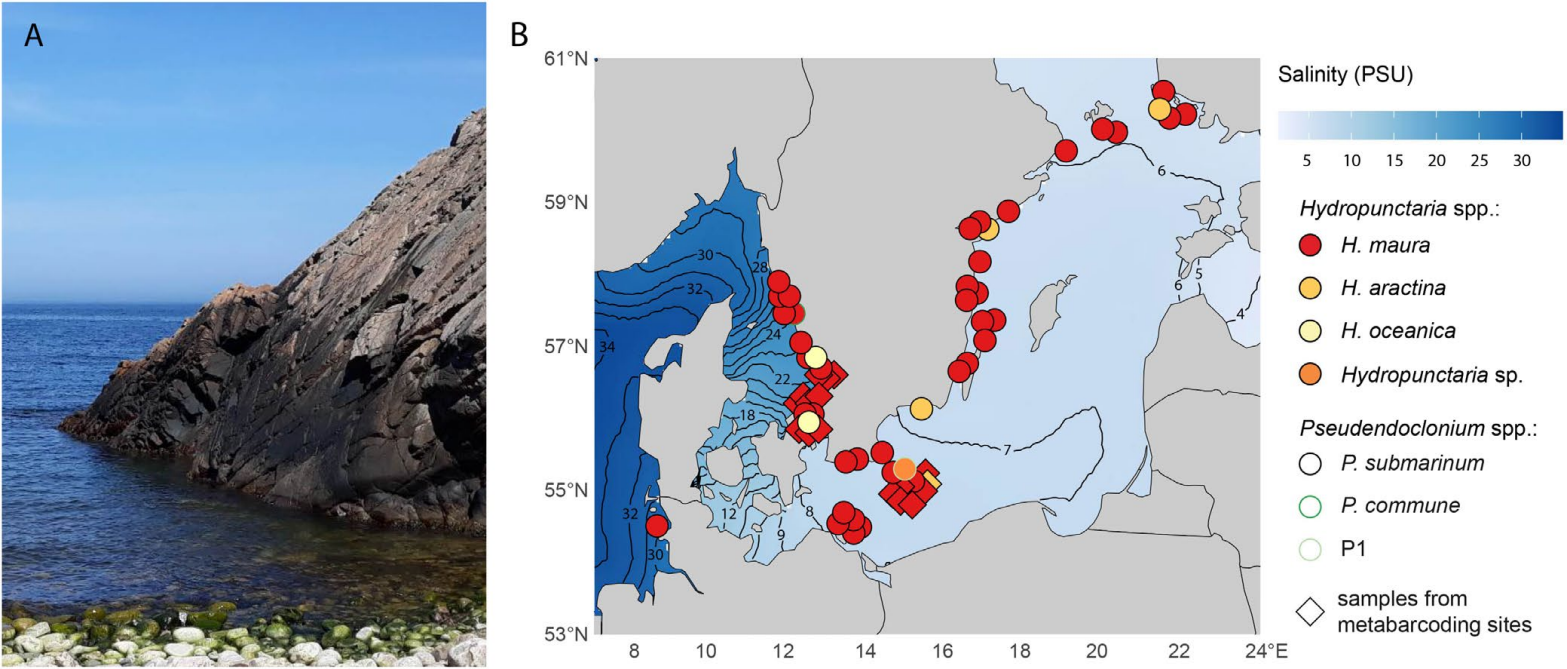
(A) So‐called black belt of *Hydropunctaria* species in Kullaberg, Sweden at low tide. Photo by I. Černajová. (B) Map of sampling sites showing surface salinity across the seas. Symbols represent collected specimens, with fill color indicating the *Hydropunctaria* species and border color representing the corresponding *Pseudendoclonium* photobionts. Squares mark the sites where free‐living algal communities were sampled and analyzed via metabarcoding.

Black crustose lichens of the genus *Hydropunctaria* dominate the mesic‐supralittoral zone (i.e., above the high‐water mark—it is not affected by regular tide but frequently washed by waves and sprayed by seawater) of rocky seashores. The most common species, *H. maura*, forms large continuous crusts that are often noticeable from a distance (Figure 1A). The few co‐occurring species can only be reliably distinguished based on microscopic examination and/or DNA sequence data (Orange 2012). *Hydropunctaria maura* has a wide ecological amplitude in terms of climatic conditions, salinity, or substrate; in Europe, it is known from Svalbard to the Canary Islands. It tolerates salt concentrations from 1 to 35 PSU (Schiefelbein 2009) and grows on various substrates, ranging from siliceous rocks to limestone (Smith et al. 2009). This raises the question of whether such ecological flexibility is linked to a broad range of its associated photobionts reflecting the environmental heterogeneity. However, despite the significance of *H. maura* on the seashore, there is surprisingly little data on the identity of its photobionts. Specifically, only five records have been published to date. These comprise three algal species of the family Kornmanniaceae, Ulvales: *Pseudendoclonium submarinum* in western Scotland, UK (SAG 2237); *P. commune* in Wales, UK and Öland, Sweden (Thüs et al. 2011); and *Halofilum ramosum* in Asturias, Spain (Gasulla et al. 2019) and Brittany, France (Darienko and Pröschold 2017).

Salinity is the primary factor determining the distribution of organisms, including lichens, on the seashore (Delmail et al. 2013). Algal strains—even within a single species—may differ in their osmoregulatory responses and halotolerance (Gasulla et al. 2019). The identity of the photobiont is thus directly linked to the lichen's realized ecological niche. Here, we sampled *Hydropunctaria* along a salinity gradient in northern Europe, expecting to find a variety of mycobiont‐photobiont associations shaped by the changing salinity. In addition, we also sampled free‐living microscopic algal communities from rocks surrounding the lichens to assess the realized associations in the context of the available photobiont pool.

## 2 Materials and Methods

### 2.1 Study Area

The Baltic Sea, a northern extension of the North Atlantic, extends from southern Denmark and northeastern Germany almost to the Arctic Circle, separating the Scandinavian Peninsula from continental Europe. It connects to the North Sea via the shallow Belt Sea and Kattegat. Due to high freshwater input, its surface salinity (Figure 1B) is much lower than that of ocean water (which is about 35 PSU—practical salinity units), ranging from about 30 PSU in the northern Kattegat to 1–2 PSU in the Bothnian Bay and Gulf of Finland.

### 2.2 Sampling

From 2019 to 2021 we sampled *Hydropunctaria* lichens from the shores of the Baltic Sea from Helsinki (Finland) westwards through the Swedish coast, including Bornholm Island (Denmark) and Rügen Island (Germany), and further along the Swedish coast of the Kattegat (Figure 1B). We also included one additional collection from the North Sea, Pellworm Island (Germany). The surface sea salinity in the sampled area ranges from 5 to 8 PSU in the Baltic Sea and from 8 to 26 PSU in the Kattegat and is about 30 in Pellworm (Bio‐ORACLE (Assis et al. 2024)). The specimens are coded BaltXX and KattXX referring to the Baltic Sea and Kattegat respectively. They are numbered westwards, with Balt01 as the easternmost specimen and Katt21 as the westernmost. The single specimen from the North Sea is coded North06. Detailed collection data are given in Table S1. Air‐dried thalli were stored at 4°C until processed.

At six collection sites (Bornholm Nexo, Bornholm Balka, Laxvik, Barsebäckshamn, Kullaberg Josefinelust, Kullaberg Ransvik; Table S1), free‐living rock‐inhabiting microscopic algal communities (available pool of photobionts) were also sampled. Rock surface that was close to the collected lichens but free of any visible lichen thalli or other organism biofilms was scraped at ten spots with a sterile spoon directly into an Eppendorf tube. The samples were immediately frozen and stored at −20°C until processed.

### 2.3 Sanger Sequencing and Phylogenetic Analyses

DNA from the lichen thalli was isolated using the CTAB protocol (Cubero et al. 1999) with minor modifications outlined in (Černajová, Steinová, et al. 2022). Nuclear ITS rDNA and mitochondrial SSU rDNA of the mycobiont were amplified using the primer pairs ITS1F (Gardes and Bruns 1993) with ITS4 (White et al. 1990) and mrSSU1 with mrSSU3R (Zoller et al. 1999), respectively. The photobionts were identified based on nuclear SSU and/or ITS rDNA. Within the family Kornmanniaceae, species can be clearly resolved using nrSSU. Although nrITS is more variable, it provides a resolution that is consistent with nrSSU (Darienko and Pröschold 2017). Similarly, its resolution is congruent with that of the hypervariable chloroplast RPL10A region (Gasulla et al. 2019). Thus, the combination of these markers is well‐suited for both reconstructing the phylogenetic position of the photobionts and capturing their intraspecific variability. The primer pairs used for amplification were 18S‐Ulvo‐F with 18S‐Ulvo‐R (Černajová, Schiefelbein, and Škaloud 2022) for nrSSU, and KlebsF (Škaloud and Rindi 2013)/newly designed ZelenyF1(5′‐CCG CCC GTC GCT CCT ACC GA‐3′) with ITS4 for nrITS. The PCR conditions were as in Černajová, Schiefelbein, and Škaloud (2022). The PCR products were purified with SPRI AMPure XP paramagnetic beads (Beckman Coulter) and sequenced by Macrogen Europe, Amsterdam, the Netherlands. For the GenBank accession numbers of the newly obtained sequences see Table S1.

The sequences were aligned with relevant sequences downloaded from GenBank separately for each marker using MAFFT v.7 (Katoh et al. 2019), applying the G‐INS‐i method and manually checked. Ambiguously aligned regions were identified using the program Gblocks v. 0.91b (Castresana 2000) applying less stringent settings and eliminated. Substitution models, estimated with JModelTest v. 2.1.4 (Darriba et al. 2012) using the Bayesian Information Criterion, are given below.

The dataset of the genus *Hydropunctaria* included all the nine currently described species (Orange 2012; Spribille et al. 2020), additional sequences available from GenBank in order to cover the genetic variability within the species, and *Staurothele fissa* as the outgroup. The final alignment contained 32 unique sequences and 473 nrITS, of which 204 were variable (V) and 162 were parsimony informative (Pi), and 633 mtSSU (97 V, 48 Pi) positions. The selected substitution models were TPM1uf + G, K80, HKY + I, and TPM3uf + G for ITS1, 5.8S, ITS2, and mtSSU, respectively.

The Kornmanniaceae datasets included all the currently recognized species with available DNA sequence data and additional undescribed *Pseudendoclonium* lineages (Černajová, Schiefelbein, and Škaloud 2022; Darienko and Pröschold 2017; Liu et al. 2019; Namba and Nakayama 2021; Škaloud et al. 2018) and consisted of 43 unique sequences, including *Ctenocladus circinatus* as the outgroup. The alignment consisted of 1581 nrSSU (215 V, 154 Pi) and 432 nrITS positions (211 V, 167 Pi). The selected substitution models were TIM3ef + I + G, TVM + G, TrNef+I, and TVM + G for SSU, ITS1, 5.8S, and ITS2, respectively.

The phylogenetic trees were inferred by maximum likelihood analyses (ML) in RAxML v. 8.2.12 (Stamatakis 2014) and Bayesian Inference (BI) in MrBayes v. 3.2.6 (Ronquist et al. 2012) using partitioned datasets. The ML bootstrap support values were calculated based on 1000 replications. In BI, two parallel Monte Carlo Markov Chain (MCMC) runs, with one cold and three heated chains, were carried out. The convergent diagnostic of the potential scale reduction factor approached 1 in all cases. The average standard deviation of split frequencies (SDSF) was 0.0011 for *Hydropunctaria* (15 million generations) and 0.0018 for Kornmanniaceae (5 million generations). All the analyses were run on the CIPRES Science Gateway v. 3.3 web portal (Miller et al. 2010).

### 2.4 Metabarcoding

DNA from the free‐living algal communities was isolated using the Fast DNA SPIN Kit for Soil (MP Biomedicals) according to the manufacturer's instructions. ITS2 amplicons from Illumina MiSeq sequencing were produced by nested PCR with the primer 1378j02 (Kantnerová and Škaloud 2025) and the primer ITS4 (White et al. 1990) in the first step and barcoded 5.8F‐Chlorophyta (Vančurová et al. 2020) and ITS4 primers in the second step. The PCRs were performed using the Q5 High‐Fidelity DNA polymerase (BioLabs Inc.); they were run in 22 and 24 cycles in the first and second step, respectively, and the conditions were as follows: initial denaturation at 98°C for 30 s, 98°C denaturation for 10 s, 52°C amplification for 45 s and 72°C elongation for 1 min, with a final 72°C extension for 2 min. Each sample was run in three replicates and three PCR negative controls (with water used instead of sample DNA) were included. The PCR products were purified with SPRI AMPure XP paramagnetic beads (Beckman Coulter), pooled equimolarly and sent for library preparation and sequencing to Fasteris (Plan‐les‐Ouates, Switzerland). Sequencing was performed on the Illumina MiSeq platform with paired‐end mode (2 × 300 bp). Quality control of the Illumina MiSeq paired‐end reads was carried out using FastQC v. 0.11.8 (Andrews 2010). Raw reads were processed according to Bálint et al. (2014), including quality filtering, paired‐end assembly, removing primer artifacts, extracting reads by barcodes, reorienting reads to 5′‐3′, demultiplexing, dereplicating, OTU clustering (this step carried out using Swarm v. 2 (Mahé et al. 2015), with distance = 3 setting), and chimera filtering. Each sample was sequenced in triplicate, and both negative controls (distilled water as template) and multiplexing controls (unused combinations of left and right barcodes) were used in library preparation. Only amplicons that were found in at least 10 reads and at least two replicates, while their sum across all the samples was higher than their sum in the negative controls, were considered. For abundance comparison, the highest number among the triplicates was used for each amplicon. Raw data are available at: http://www.ncbi.nlm.nih.gov/bioproject/1114736.

The amplicons were identified by BLAST searches in SEED2 (Větrovský et al. 2018), and only Viridiplantae sequences were further processed. To identify the algae present in the free‐living communities, the obtained sequences were aligned with the closest BLAST matches (based on % similarity and *E* value) and a ML tree was constructed as above. Based on the resulting phylogenetic tree, each amplicon was assigned to a taxonomic unit.

### 2.5 Data Visualization

All the plots and the map were built in the free software R v. 4.1.0 (R Core Team 2021) using the packages ggplot2 (Wickham 2016), circlize (Gu et al. 2014), raster (Hijmans 2025), rnaturalearth (Massicotte and South 2023), rnaturalearthdata (South et al. 2024), sdmpredictors (Bosch and Fernandez 2023), and sf (Pebesma and Bivand 2018). The surface salinity data were downloaded from Bio‐ORACLE (https://bio‐oracle.org/; (Assis et al. 2024)).

## 3 Results

Both mycobiont and photobiont sequences were successfully obtained from 62 samples; no photobiont sequence was obtained from a specimen of *H. oceanica* (Katt15).

### 3.1 Mycobiont Diversity

**FIGURE 2:**
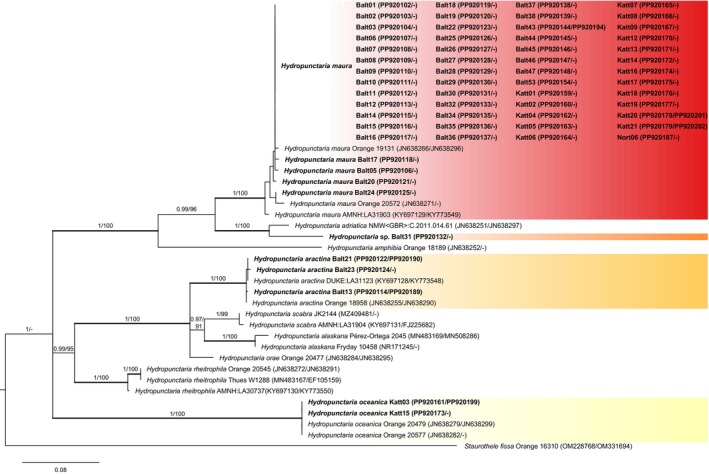
Phylogeny of the genus *Hydropunctaria* based on maximum likelihood (ML) of concatenated mtSSU and nrITS DNA. Values at nodes show statistical support calculated by MrBayes posterior‐node probability (PP)/ML bootstrap. Only statistical supports with bootstrap values > 60 are shown. GenBank accession numbers are given in brackets (nrITS/mtSSU). Newly obtained sequences are in bold. Scale bar represents the expected number of substitutions per site.

**FIGURE 3:**
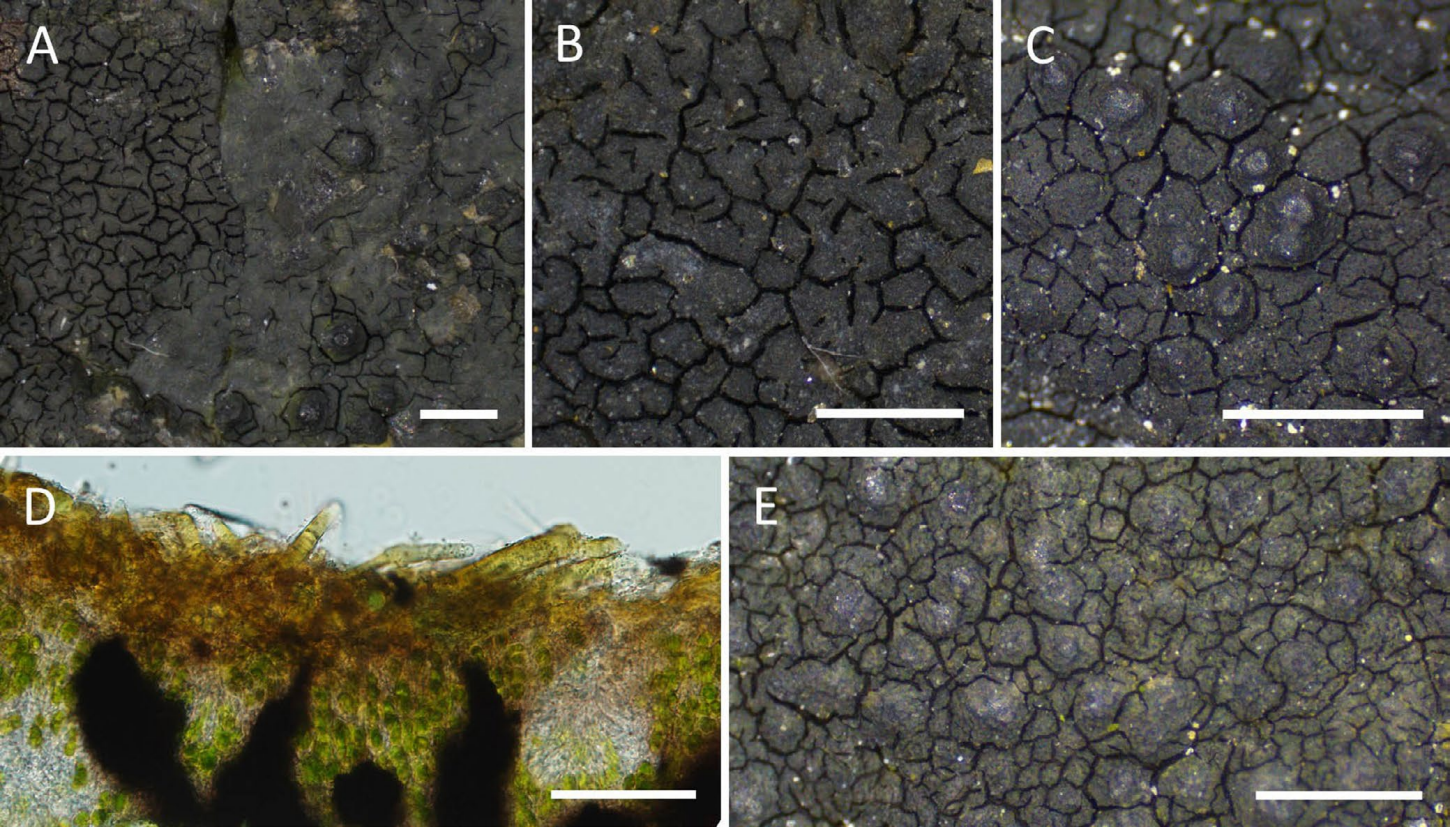
*Hydropunctaria* species. (A) *H. maura* (left) and *H. aractina* (right) when growing together; (B) *H. maura* alone; (C) *H. aractina* alone; (D) Cyanobacteria on the thallus surface disguising cortical pigments; (E) *H. oceanica* . Scale bars represent 1 mm (A–C, E) and 100 μm (D). Photos by I. Černajová.

The phylogeny of *Hydropunctaria* (Figure 2) revealed that in addition to the dominant *H. maura* , *H. aractina* (4 specimens), *H. oceanica* (2 specimens), and a novel lineage related to *H. adriatica* (Balt31) were also present. *H. aractina* had previously been known only from northern Norway and differs from *H. maura* in dull green cortical pigments and thallus thickness; however, there are great overlaps between the two species (Orange 2012). As stated in the *H. aractina* description (Orange 2012), the distinction between the species was obvious when found together (Figure 3A) but isolated thalli were virtually impossible to distinguish (Figure 3B, Figure 3C). This was partly because the thalli were often overgrown by filamentous cyanobacteria, which completely disguised the cortical pigments (Figure 3D). Additionally, only a few spores were found. In our dataset, *H. aractina* was only found in the Baltic Sea (Figure 1B, Table S1). *H. oceanica* was previously only known from the British Isles; in our study, we found it exclusively in the Kattegat region (Figure 1B, Table S1). It is expected to differ from *H. maura* primarily due to the conspicuously protruding perithecia (Orange 2012); however, this characteristic was not observed in our collections (Figure 3E).

### 3.2 Photobiont Diversity

**FIGURE 4:**
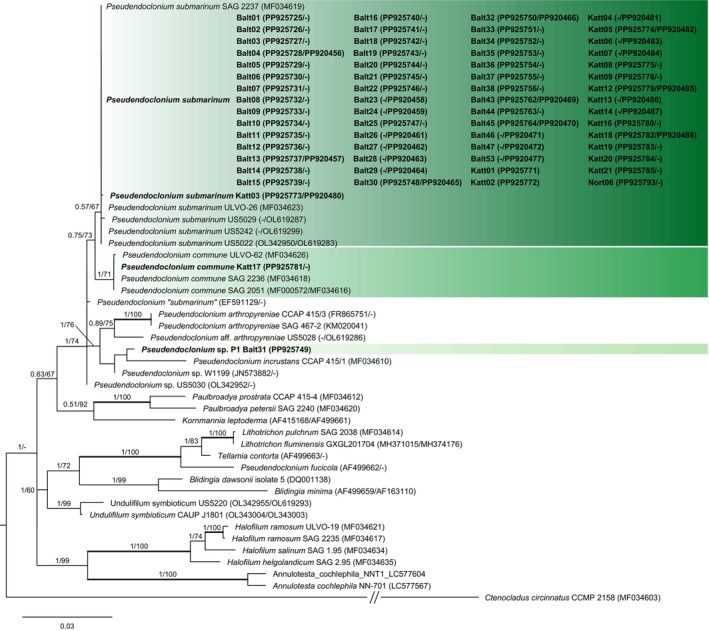
Phylogeny of the family Kornmanniaceae based on maximum likelihood (ML) of concatenated nrSSU and nrITS DNA. Values at nodes show statistical support calculated by MrBayes posterior‐node probability (PP)/ML bootstrap. Only statistical supports with bootstrap values > 60 are shown. Thick branches represent nodes fully supported by both analyses. GenBank accession numbers are given in brackets (nrSSU/ITS). Newly obtained sequences are in bold. Scale bar represents the expected number of substitutions per site.

Sixty photobionts were identified as *Pseudendoclonium submarinum*, one as *P. commune* and another as belonging to a previously unknown lineage, labeled *Pseudendoclonium* sp. P1 here, whose phylogenetic placement was not supported (Table S1, Figure 4). All the obtained *P. submarinum* nrSSU sequences were identical, while three nrITS genotypes were detected. The most abundant genotype was found in 19 samples. The second genotype—differing by a single nucleotide—was found only in sample Katt03. The third genotype was shared by four samples (Balt24, Balt25, Katt13, Katt14) and also differed by one nucleotide from the most abundant genotype; however, this substitution occurred within a region that was eliminated from the analysis due to ambiguous alignment, and thus, it is not visible in the final phylogeny.

### 3.3 Symbiont Pairings

**FIGURE 5:**
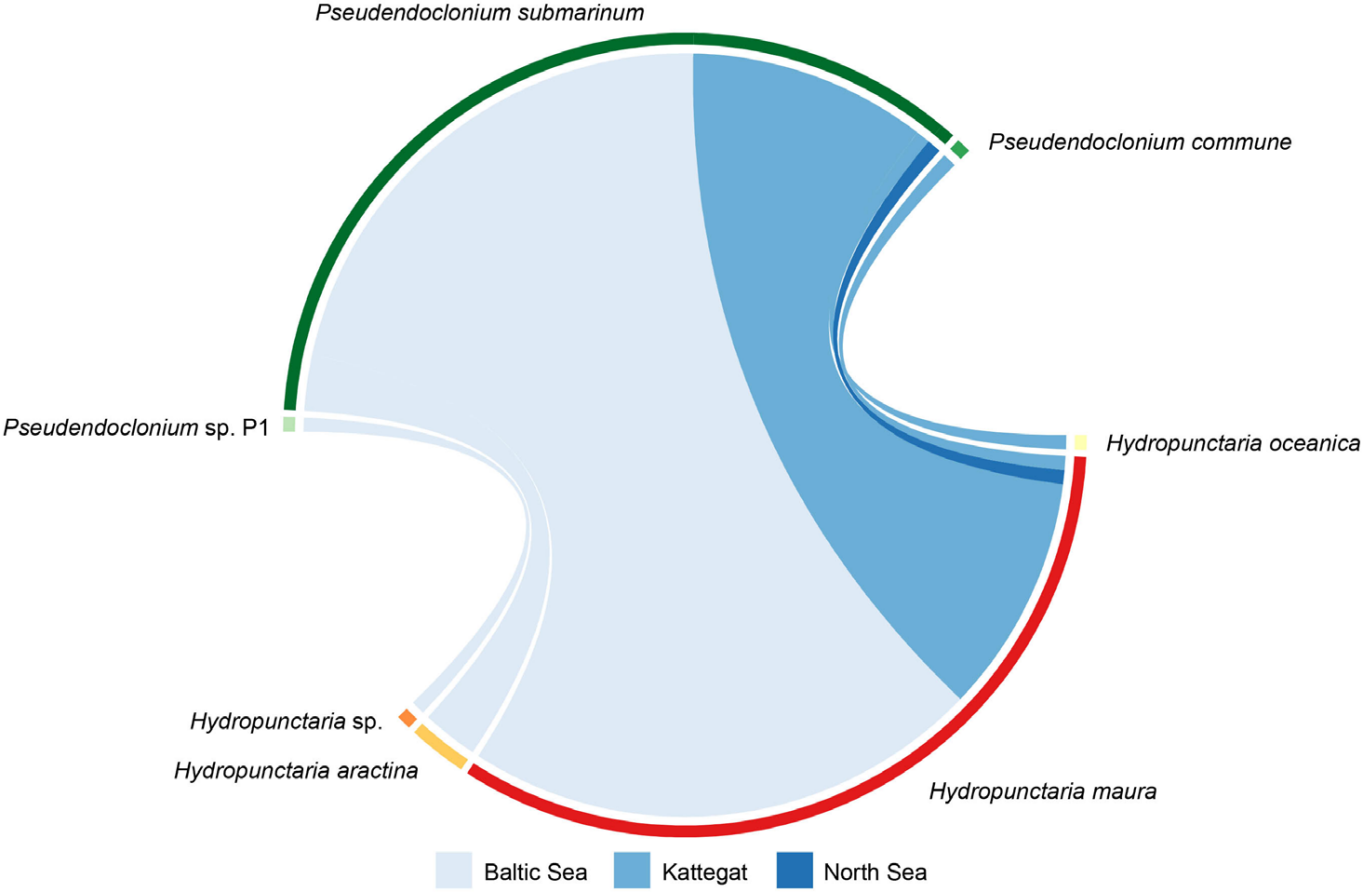
Mycobiont‐photobiont association network. Link widths are proportional to the number of samples in the association. Links are colored according to salinity zones: the Baltic Sea (5–8 PSU), Kattegat (8–26 PSU), and North Sea (26–35 PSU).

*Hydropunctaria aractina* and *H. oceanica* associated with *P. submarinum* only, *Hydropunctaria* sp. (Balt31) with *Pseudendoclonium* sp. P1, and *H. maura* with *P. submarinum* (55 samples) and *P. commune* (one sample, Figure 5). Interestingly, the *H. maura–P*. *commune* (Katt17) association was recorded at a site (Fiskebäck, Kattegat, Sweden) where the common association *H. maura–P. submarinum* (Katt18) was also found. However, they were collected from different microsites—Katt18 (*P. submarinum*) from a rock surface directly facing the sea, and Katt17 ( *P. commune* ) from a more sheltered surface on the landward side of a rock projecting into the sea.

### 3.4 Available Photobiont Pool

**FIGURE 6:**
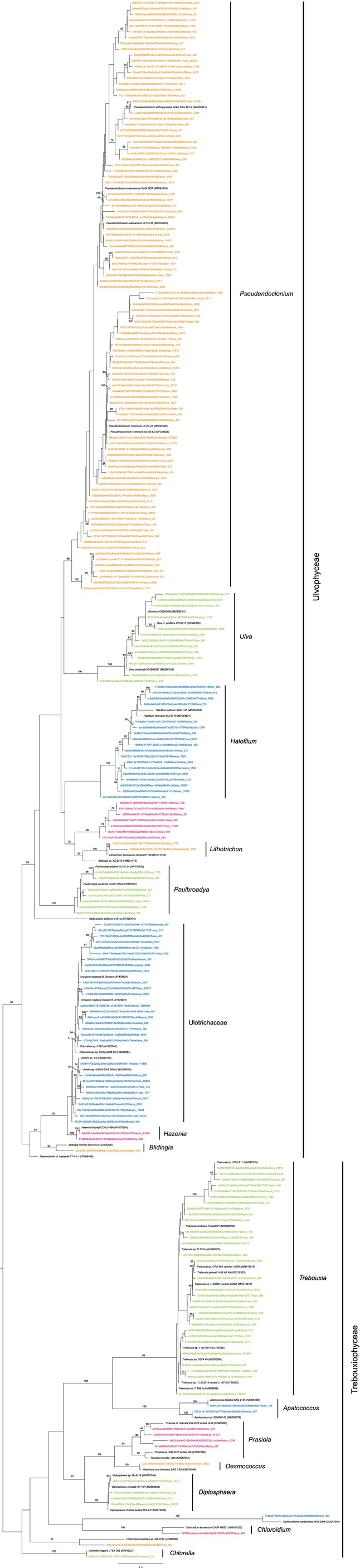
Phylogenetic tree of Ulvophyceae and Trebouxiophyceae based on Maximum Likelihood (ML) of ITS2 rDNA obtained by Illumina metabarcoding of the free‐living algal communities (in color) together with GenBank reference sequences. Only ML bootstrap values > 60 are shown. Scale bar represents the expected number of substitutions per site.

**FIGURE 7:**
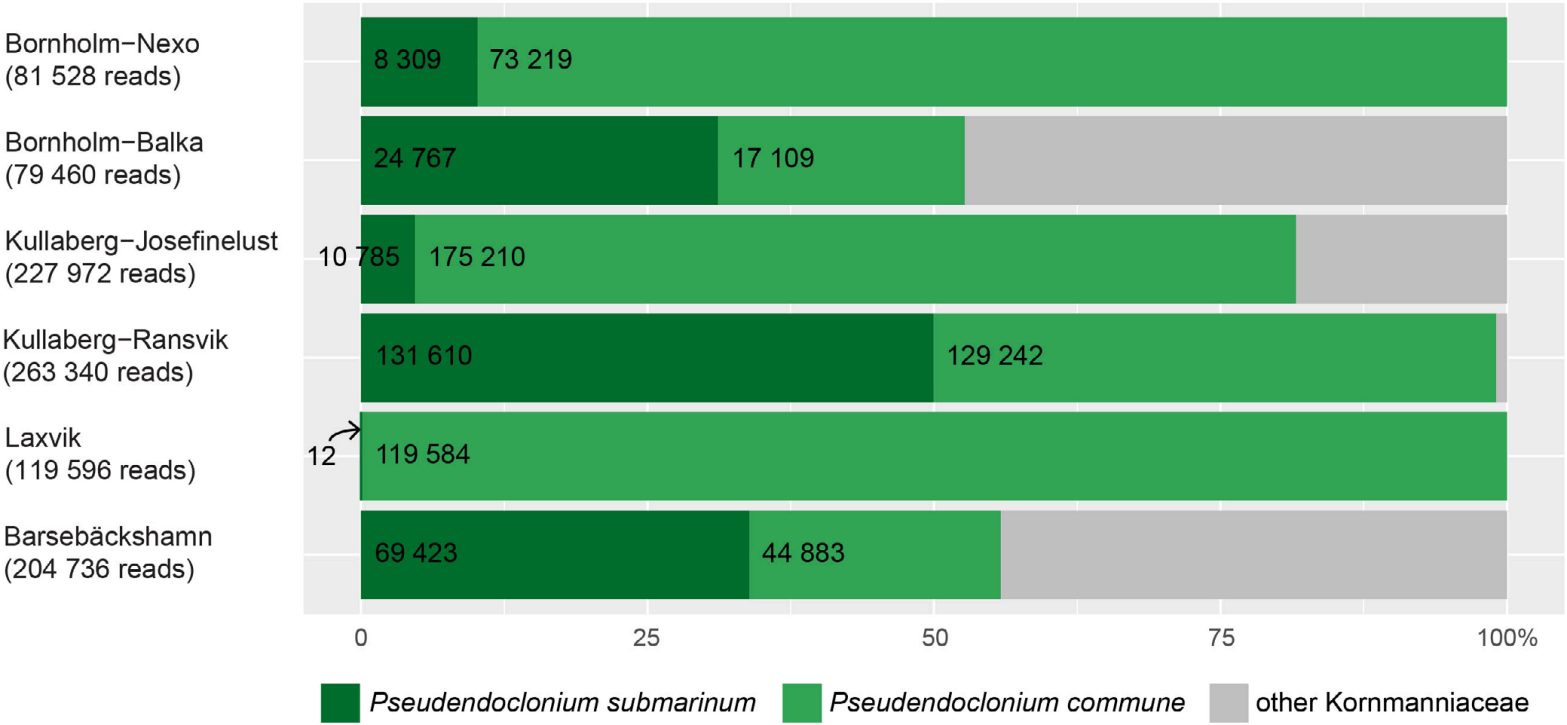
Proportion of *P. submarinum* and *P. commune* within the family Kornmanniaceae in the free‐living algal communities, as inferred by metabarcoding. For each locality, the number of reads assigned to each species and the total number of reads belonging to Kornmanniaceae are shown.

A total of 7,094,927 reads passed the bioinformatic filtering, resulting in 124 amplicons. Their best Blast matches are given in Table S2. The Viridiplantae free‐living rock‐inhabiting algal communities were dominated by Ulvophyceae and Trebouxiophyceae (Figure 6) and photobiont genera made up a significant portion of the overall community. A list of the detected genera together with their read numbers at each site is given in Table S3. We focused specifically on the family Kornmanniaceae, which represents potential associates of *H. maura* . Among them, the genus *Pseudendoclonium* was the most abundant. *Halofilum ramosum*, another known symbiont of *H. maura* , was not detected; however, various sequences belonging to the genus *Halofilum* were found. These probably represent yet unknown taxa. The ratio of *P. submarinum* and *P. commune* —the two *H. maura* photobionts identified in our study—to the remaining Kornmanniaceae sequences at each site, along with their total read counts per locality, is illustrated in Figure 7. *P. commune* was available to *H. maura* at each of these sites, at some of them presumably in higher abundances than *P. submarinum*. This fact highlights the high selectivity of *H. maura* . Note that at Laxvik, *P. submarinum* was also detected but only in 12 reads.

## 4 Discussion

Partner breadth directly shapes the mutualistic niche breadth, and the ability to associate with multiple partners is particularly advantageous in heterogeneous and fluctuating environments (Batstone et al. 2018). This strategy of partner diversity aligning with environmental conditions is widespread across mutualistic systems—for example, it has been documented in lichens, corals, pollinators, endophytic fungi–plants, and mycorrhizal fungi–plant host associations (Albrecht et al. 2012; Gerz et al. 2018; Peay 2016; Rodriguez et al. 2008; Rolshausen et al. 2020; Silverstein et al. 2012). Contrarily, specialization to a single symbiont only rarely results in broad ecological niches (Batstone et al. 2018; Zarate et al. 2024), with the stable association of the coral *Pocillopora verrucosa* with *Symbiodinium microadriaticum* along a cross‐shelf and depth gradient providing an example (Ziegler et al. 2015).

The stable association between *Hydropunctaria maura* and *Pseudendoclonium submarinum* found along the salinity gradient studied here provides further evidence that relying on a single symbiont with a wide ecological amplitude can support a broad ecological niche. Algal tolerance to specific salinity levels—and the breadth of the salinity range tolerated—varies greatly between species and is one of the main factors driving turnover in algal communities (Olofsson et al. 2020; Pinseel et al. 2022; Talarski et al. 2016; Telesh et al. 2013). Tolerance to a wide range of salinities is a necessary capability of organisms—including lichens, algae, or animals—to thrive in the supralittoral zone, where hypoosmotic (caused by rainfall) and hyperosmotic (resulting from wave action, seawater spray, or desiccation) conditions fluctuate frequently (Jacob et al. 1991; Leeuwis and Gamperl 2022). The higher the seawater salinity, the greater the fluctuations at a given site.

Accordingly, *P. submarinum* is a euryhaline species (tolerating a wide range of salinities), although data on its distribution and ecological preferences are limited. It has been reported as a lichen photobiont from western Scotland (SAG 2237) and from the west coast of Chile (Černajová, Schiefelbein, and Škaloud 2022), both with salinities of about 35 PSU (i.e., the upper range of salinity values commonly found along the world's seashores). Its type locality lies in southern Norway, with a salinity of about 21 PSU, where it was found as a free‐living alga (Mullins 2007). Additionally, it has also been recorded as a free‐living aerophyte on Gran Canaria, Spain (BEA 0994B). We assume that this exceptional breadth of salinity tolerance, underscored by salinities from 5 to 30 PSU in this study, plays a key role in facilitating the broad ecological niche occupied by *H. maura* . Alternatively, *P. submarinum* may comprise multiple strains that differ in salinity‐related traits but remain unrecognized based on the markers used. However, no data are currently available to support or reject this hypothesis.

Stability of a mutualistic association may also be shaped by the absence or unavailability of diverse compatible symbionts in the environment (Batstone et al. 2018). In our study, not only did the *H. maura—P. submarinum* association persist along the salinity gradient, but it was also maintained despite the apparent unavailability of the photobiont in the surrounding environment. Recruitment of photobionts *de novo* at the beginning of thallus formation remains one of the fundamental questions in lichen biology. The recent accessibility of HTS methods brings a powerful tool into the debate. Yet, metabarcoding studies of algal diversity in the environment are scarce (e.g., Frey et al. 2013; Lutz et al. 2015; Rippin et al. 2018) and a single one has focused on the diversity of photobionts so far (Vančurová et al. 2020). It confirmed the presence of lichen photobionts in soil and also showed high selectivity of *Stereocaulon* lichens, as algae commonly found in soil were rare in lichen thalli and vice versa (Vančurová et al. 2020). A comparable picture emerges from the data of Chrismas et al. (2021) who showed high selectivity even for accessory photobionts in the case of the intertidal *L. pygmaea* . Comparing the endothallic and epithallic communities of algae and cyanobacteria, they found that the lineages most abundant within the lichen thalli were rare on their surface and vice versa (Chrismas et al. 2021). Here, we report a similar pattern. The genera of photobionts, including *Trebouxia*—the most common lichen photobiont genus, sometimes still believed to be absent from outside of lichens (summarized by Veselá et al. 2024), made up a significant portion of the whole Viridiplantae community. Additionally, *P. commune* , a compatible photobiont of *H. maura* , was commonly more abundant within the available photobiont pool than *P. submarinum*. Thus, the stability in symbiont pairing cannot be attributed to the absence of other potential partners.

Beyond salinity, associations with multiple photobionts may still broaden the niche of *H. maura* along niche axes. Previous records of its association with *Halofilum ramosum* in France and northern Spain (Darienko and Pröschold 2017; Gasulla et al. 2019) and with *Pseudendoclonium commune* in Wales, UK (Thüs et al. 2011) suggest that these axes may correspond to climatic variables. Complementarily, they may relate to environmental factors, such as seawater inundation, sun exposure, and desiccation, that exhibit steep gradients along the littoral zone and lead to pronounced vertical zonation among organisms, including lichens and their photobionts (Chappuis et al. 2014; Fletcher 1973, 1975; Gasulla et al. 2019; Ortiz‐Álvarez et al. 2015; Tindall‐Jones et al. 2023). Additionally, specific site conditions and microhabitat characteristics also play a role in shaping these zones (Fletcher 1973). The single recorded association of *H. maura* with *P. commune* in this study may reflect the influence of microhabitat. It was found at a site where the *H. maura–P. submarinum* association also occurred but in a different microhabitat. Further studies are needed to address these specific effects.

In summary, the case study of *H. maura–P. submarinum* shows that a stable association between generalist symbionts—generalists in terms of ecological amplitude rather than partner specificity—may facilitate occupation of an ecological niche along a whole niche axis (the whole range of salinity here). Complementarily, association with different symbionts may broaden the niche along its other axes, corresponding to other environmental factors. Our study also highlights the presence of lichen photobionts in free‐living algal communities and points to selective mechanisms beyond partner availability alone. Further research based on high‐resolution biodiversity data will be needed to clarify the role of symbiont diversity in shaping the exceptionally broad ecological niche of *H. maura* .

## Author Contributions

**Ivana Černajová:** conceptualization (equal), data curation (equal), formal analysis (lead), investigation (equal), methodology (equal), visualization (lead), writing – original draft (lead), writing – review and editing (equal). **Jana Schmidtová:** conceptualization (equal), data curation (equal), formal analysis (supporting), investigation (equal), methodology (equal), writing – original draft (supporting), writing – review and editing (equal). **Ulf Schiefelbein:** conceptualization (supporting), investigation (equal), writing – review and editing (equal). **Francesco Dal Grande:** data curation (equal), formal analysis (supporting), writing – review and editing (equal). **Pavel Škaloud:** conceptualization (equal), formal analysis (equal), funding acquisition (lead), investigation (equal), methodology (equal), visualization (supporting), writing – review and editing (equal).

## Funding

This work was supported by the Czech Science Foundation GA ČR 24‐10510 K.

## Conflicts of Interest

The authors declare no conflicts of interest.

## Supporting information


**Table S1:** Collection data, symbiont identity, and GenBank accession numbers. Localities where free‐living algal communities were also sampled are highlighted in blue.
**Table S2:** Taxonomic assignment and best BLAST match for each amplicon obtained from metabarcoding of free‐living algal communities.
**Table S3:** Genera detected by metabarcoding in free‐living algal communities and the number of reads per locality. For the family Ulotrichaceae, genera could not be reliably distinguished based on ITS2 sequences; therefore, read counts were summed at the family level.

## Data Availability

All the newly generated sequences are publicly available from GenBank. The accession numbers for sequences obtained by Sanger sequencing are given in Table S1 and in Figures 2 and 4. Sequences obtained by Illumina MiSeq are available at http://www.ncbi.nlm.nih.gov/bioproject/1114736.
